# Differential expression of the catalytic subunits for PP-1 and PP-2A and the regulatory subunits for PP–2A in mouse eye

**Published:** 2008-04-21

**Authors:** Wen-Bin Liu, Yong Li, Lan Zhang, He-Ge Chen, Shuming Sun, Jin-Ping Liu, Yun Liu, David Wan-Cheng Li

**Affiliations:** 1 Key Laboratory of Protein Chemistry and Developmental Biology of National Education Ministry of China, College of Life Sciences, Hunan Normal University, Changsha, Hunan, China; 2 Department of Biochemistry & Molecular Biology, College of Medicine, Nebraska Medical Center, Omaha, NE; 3 Department of Ophthalmology & Visual Sciences, College of Medicine, Nebraska Medical Center, Omaha, NE

## Abstract

**Purpose:**

Reversible protein phosphorylation is a fundamental regulatory mechanism in all biologic processes. Protein serine/threonine phosphatases-1 (PP-1) and 2A (PP-2A) account for 90% of serine/threonine phosphatase activity in eukaryote cells and play distinct roles in regulating multiple cellular processes and activities. Our previous studies have established the expression patterns of the catalytic subunits for PP-1 (PP-1cs) and PP-2A (PP-2Acs) in bovine and rat lenses. In the present study, we have determined the expression patterns of PP-1cs (PP-1α and PP-1β) and PP-2Acs (PP-2Aα and PP-2Aβ) in the retina and cornea along with the ocular lens of the mouse eye. Moreover, since the function of PP-2A is largely relied on its regulatory subunits, we have also analyzed the expression patterns of the genes encoding the scaffold A subunits of PP-2A, PP2A-Aα and PP2A-Aβ, and the regulatory B family subunits of PP-2A, PP2A-Bα, PP2A-Bβ, and PP2A-Bγ. In addition, we have also demonstrated the differential protections of PP-1 and PP-2A in mouse lens epithelial cell line, αTN4–1, against oxidative stress-induced apoptosis.

**Methods:**

Total RNAs and proteins were extracted from the retina, lens epithelium, lens fiber cells, and cornea of the mouse eye. Reverse transcription polymerase chain reaction (RT–PCR) and real time PCR were used to detect the mRNA expression. Western blot and immunohistochemistry analysis were applied to examine the protein expression and distribution. Stable clones of αTN4–1 cells expressing either PP-1α or PP-2Aα were used to analyze the differential protections against oxidative stress-induced apoptosis.

**Results:**

PP-1 is more abundant than PP-2A in the mouse eye. The catalytic subunits for PP-1 and PP-2A display similar expression patterns in the retina and cornea but much reduced in the lens. The mRNAs for all five isoforms of PP2A-A and PP2A-B subunits are highly expressed in the retina, but only three out of the five mRNAs are expressed in the cornea. In the ocular lens, only PP2A-Aβ and PP2A-Bγ mRNAs are clearly detectable. The A and B subunit proteins of PP-2A are highly expressed in the retina and cornea but are much reduced in the ocular lens. PP2A-Aα/β are differentially distributed in the mouse retina.When transfected into mouse lens epithelial cells, αTN4–1, PP-1α and PP-2Aα display differential protection against oxidative stress-induced apoptosis.

**Conclusions:**

Our results lead to the following conclusions regarding PP-1 and PP-2A in mouse eye: 1) PP1 is a more abundant phosphatase than PP-2A; 2) both PP-1 and PP-2A may play important roles, and the functions of PP-2A appear to be highly regulated by various regulatory subunits; and 3) the genes encoding PP-1α/β, PP-2Aα/β, PP-2A-Aα/β, and PP-2A-B α/β/γ are all differentially expressed.

## Introduction

Protein phosphorylation and dephosphorylation are the most important regulatory mechanisms governing many aspects of biology [1]. Phosphoproteome studies have revealed that phosphorylation and dephosphorylation modulate functions of more than one third of the total cellular proteins [2]. Thus, it is conceivable that protein phosphorylation and dephosphorylation can regulate many different biologic processes such as gene expression, cell cycle progression, differentiation, transformation, apoptosis, neuronal transmission, and many other cellular activities [1-6].

In eukaryotes, dephosphorylation at serine/threonine residues are executed by four major protein phosphatases, phosphatase-1 (PP-1), phosphatase-2A (PP-2A), phosphatase-2B (PP-2B), and phosphatase-2C (PP-2C) [5,6] and several minor phosphatases including phosphatase-4 (PP-4), phosphatase-5 (PP-5), phosphatase-6 (PP-6), and phosphatase-7 (PP-7) [5,6]. Among these different serine/threonine phosphatases, PP-1 and PP-2A accounts for 90% of the intracellular protein serine/threonine phosphatase activities [6].

Although more than 90 PP-1 interacting proteins or targeting proteins have been identified or predicted, the functional enzyme of PP-1 exists as a single catalytic subunit [6]. On the other hand, the holoenzyme of PP-2A is a heterotrimer, which consists of a scaffold A subunit tethering a catalytic C subunit, and a regulatory B subunit [7]. Both A and C subunits exist in α and β isoforms encoded by different genes. However, for the B subunit, at least 16 genes have been identified encoding four subfamilies of the regulatory subunits (B, B’, B,” and B’”) [7]. The B family of regulatory subunits contains several different isoforms including α, β, and γ [7].

Both PP-1 and PP-2A play important roles in the eye. For example, the retinoblastoma protein (Rb) acts as a tumor suppressor and is also important for eye development [8]. Conditional inactivation of Rb through the overexpression of viral gene E7 in the ocular lens leads to microphthalmia and cataracts [9]. PP-1 directly dephosphorylates Rb to modulate its function [10]. During eye development, one of the B family regulatory subunits of PP-2A was found participating in both IGF/PI3K/Akt and hedgehog signaling pathways to regulate the separation of the eye field and the early induction of eye development [11]. PP-2A was also found to participate in the signaling cross-talk of the mitogen-activated protein kinase (MAPK) pathways in the cornea [12].

We have previously shown that PP-1 is a predominant phosphatase in the ocular lens [13]. The inhibition of PP-1 but not PP-2A by okadaic acid induces apoptosis of the treated rabbit and rat lens epithelial cells [14,15]. In exploring the possible molecular mechanism by which PP-1 promotes survival of lens epithelial cells, we have recently demonstrated that PP-1 can directly dephosphorylate p53, a tumor repressor and a master regulator of apoptosis [16] whose inactivation causes lens cataractogenesis [9]. Through dephosphorylation of p53 at serine residue 15 and 37, PP-1 downregulates the transcriptional and apoptotic activities of p53 [16]. More recently, we have demonstrated that PP-1 is a major phosphatase that dephosphorylates Pax−6 to modulate its function in regulating brain and eye development [17].

To further study protein phosphatase–1 and protein phosphatase–2A in the eye, we have compared the differential expression patterns of the α and β catalytic subunits for PP-1 and PP-2A in the retina, lens epithelium, lens fiber cells, and cornea of the mouse eye. Furthermore, we have analyzed the expression patterns of five different isoforms of PP-2A regulatory subunits, PP2A-Aα, PP2A-Aβ, PP2A-Bα, PP2A-Bβ, and PP2A-Bγ in the above four tissues. Our results demonstrate that PP-1 is a more abundant phosphatase than PP-2A in all four tissues of the mouse eye. Both PP-1 and PP-2A may play important roles in the mouse eye, and the functions of PP-2A appear to be highly regulated in the mouse retina and cornea. The genes encoding PP-1α/β, PP-2Aα/β, PP2A-Aα/β, and PP2A-Bα/β/γ are all differentially expressed in the four different tissues of the mouse eye to ensure their proper functions. In addition, when introduced into lens epithelial cells, αTN4–1, PP-1 and PP-2A protect lens epithelial cells against oxidative stress-induced apoptosis.

## Methods

### Animals

Mice used in this study were handled in compliance with the “Guide for the Care and Use of Laboratory Animals” (National Academy Press, Washington, DC). Four-week-old mice and 15.5-day-old embryonic mice were obtained from the Laboratory Animal Center of Hunan Normal University, Changsha, Hunan, China. A total of 84 four-week-old mice were used for the collection of corneal, retinal, lens epithelium, and lens fiber cells. These samples were used for extraction of total RNA and total proteins. Nine 15.5-day-old embryonic mice were used for immunohistochemistry.

### Preparation of total RNAs from various tissues of mouse eye

For collection of mouse eye tissues, mice were euthanatized by CO_2_ inhalation. The eyeballs were removed, and various components of eye tissues were carefully dissected by a posterior approach [18]. The retina, lens capsule/epithelial cells, lens fiber cells, and cornea were removed immediately and transferred into Eppendorf tubes containing 500 µl RNA extraction buffer (Trizol, BRL CAT# 15596–026; Gibco, Gaithersburg, MD) and were homogenized on ice with an Eppendorf tube micropestle (Brinkmann Instruments Inc., Westbury, NY). The remaining procedures of RNA extraction were the same as previously described [18,19].

### Reverse transcription-linked polymerase chain reaction

Reverse transcription was conducted using a kit from Invitrogen (#18085–019; Invitrogen, Carlsbad, CA) as previously described [13,17,19]. Briefly, 3 µg of total RNA were used in a total reaction volume of 25 µl. For polymerase chain reaction (PCR) amplification, all the primers used are listed in Table 1. Two microliters of the reverse transcription reaction mixture were used for the PCR reaction. For PCR, the primers for β-actin and for each specific gene mentioned in Table 1 were added at the same time for a total of 30 cycle amplification. Each cycle was run with the following program: denaturing at 94 °C for 30 s; annealing at 52 °C for 30 s; and chain extension at 72 °C for 1 min. At the end of each reaction, the PCR products were separated by agarose gel (1.5%) electrophoresis and photographed under ultraviolet (UV) illumination.

**Table 1:** Oligo Primers used for RT–PCR studies.

**Primer name**	**Oligo primer sequence**
PP-1α (+)	5′-GCAAGCAGTCTTTGGAGACC-3′
PP-1α (−)	5′-GCCCCAAAGGTAAAGGAGAC-3′
PP-1β (+)	5′-TGTCATGGAGGACTGTCACC-3′
PP-1β (−)	5′-CGGTGGATTAGCTGTTCGAG-3′
PP-2Aα (+)	5′-CARGAGGTTCGATGTCCAGT-3′
PP-2Aα (−)	5′-TGACCACAGCAAGTCACACA-3′
PP-2Aβ (+)	5′-GTCTGTGGAGATGTGCATGG-3′
PP-2Aβ (−)	5′-TCCAGGGCTCTTATGTGGTC-3′
β-actin (+)	5′-CACTGCCGCATCCTCTTCCT-3′
β-actin (−)	5′-ATGCCTGGGTACATGGTGGT-3′
PP2A-Aα (+)	5′-TCTAACCTGGCCTCTGACG-3′
PP2A-Aα (−)	5′-TGACATGTTGGTTGGCATCT-3′
PP2A-Aβ (+)	5′-ACTGGCTGAAGATGCCAAGT-3′
PP2A-Aβ (−)	5′-CACATTGAAGCGGACATTTG-3′
PP2A-Bα (+)	5′-ATCAAGCCTGCCAATATGGA-3′
PP2A-Bα (−)	5′-TCAGACCCATTCCAACAACA-3′
PP2A-Bβ (+)	5′-TCAGACCCATTCCAACAACA-3′
PP2A-Bβ (−)	5′-AGCCTTCTGGCCTCTTATCC-3′
PP2A-Bγ (+)	5′-CCCAGAAGAGGATGAACCAA-3′
PP2A-Bγ (−)	5′-AACTGCACGACACAGTACGC-3′

### Quantitative real-time reverse transcription-linked polymerase chain reaction

Real-time PCR was conducted as previously described [20] with all the primers used in this study listed in Table 2. Briefly, the reverse transcription was conducted using 1 µg total RNA, 5 U AMV polymerase, 1 µM oligo d(T)15 in a final volume of 20 µl. Real-time PCR reaction was conducted in 20 µl containing 1.5 µl reverse transcription polymerase chain reaction (RT–PCR) mixture, 10 µl 2X SYBR Green PCR Master Mix (ABI, Foster City, CA), and 2x10^−4^ M of specific primers. The reaction was performed using the Prism 7500 Sequence Detection System (ABI) with the following conditions: pretreatment at 50 °C for 2 min; 95 °C for 10 min, followed by a total of 40 cycles: denaturing at 95 °C for 15s; and annealing and extension at 61 °C for 45s. The real-time PCR results were analyzed with SDS 7300/7500 software (ABI).

### Preparation of total proteins from various ocular tissues of the mouse eye

After dissection of various components of the mouse eye, they were transferred into an Eppendorf tube containing 200 µl of extraction buffer (50 mM Tris-HCl, pH 7.0; 0.1% β-mercaptoethanol; 0.1 mM EDTA, 0.1 mM EGTA, 2 mM leupeptin, 1 mM PMSF, 1 mM benzamidine-HCl, 2 mM DTT, 0.5% Triton X-100) and homogenized on ice with an Eppendorf tube micropestle (Brinkmann Instruments Inc.). The remaining procedures were the same as previously described [21].

**Figure 1:**
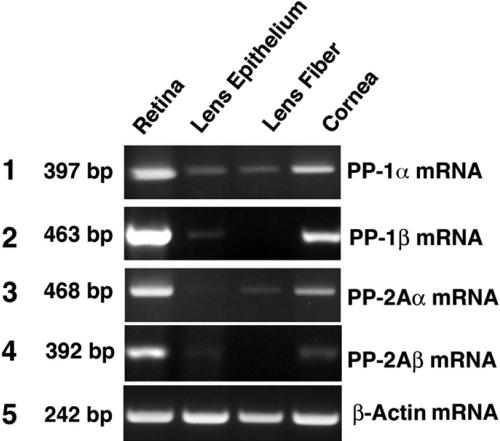
RT–PCR to detect the mRNA levels for the catalytic subunits of PP-1α, PP-1β, PP-2Aα, and PP-2Aβ in the retina, lens epithelium, lens fiber, and cornea of the mouse eye. RT–PCR was conducted as previously described [13]. The primers used in the present studies are described in Table 1. The PP-1 or PP-2A primers and the primers for β-actin were added to the reactions at the same time. The 397 bp DNA band for the PP-1α mRNA, the 463 bp DNA band for PP-1β mRNA, the 468 bp DNA band for the PP2Aα mRNA, and the 392 bp DNA band for PP-2Aβ mRNA were expressed at the highest levels in the retina, at reduced levels in the cornea, and at much lower levels in the lens epithelium and fiber cells. In addition, PP-1β was hardly detectable in the lens fiber cells. As an internal control, a β-actin DNA band of 242 bp is also included as comparison.

### Western blot analysis

Western blot analysis was conducted as previously described [21]. Briefly, 50 µg of total proteins from various ocular tissues of the mouse eye were separated by 10% SDS–PAGE and transferred into supported nitrocellulose membranes (Gibco BRL, Gaithersburg, MD). The protein blots were blocked with 5% milk in TBS (10 mM Tris, pH 8.0; 150 mM NaCl) overnight at 4 °C. Each blot was then incubated with an anti-PP-1α/β, PP-2Aα/β, PP2A-Aα/β, PP2A-Bα/β/γ antibody (primary antibody from Cell Signaling Inc. [Beverly, MA] and Santa Cruz Biotechnology [Santa Cruz, CA]) at a dilution of 1:500 or 1:1000 in 5% milk prepared in TBS for 60 min at 4 °C with mild shaking. After three 15 min washes with TBS-T (TBS with 0.05% Tween-20), each blot was incubated with a secondary antibody (anti-mouse and rabbit IgG from Amersham [Louisville, CO] or anti-goat IgG from Santa Cruz Biotechnology) at a dilution of 1:1000 for 45 min. After two washes with TBS-T followed by another two washes with TBS (15 min each), the PP-1α/β, PP-2Aα/β, PP2A-Aα/β, and PP2A-Bα/β/γ proteins were detected with an enhanced chemiluminescence detection kit according to the instruction manual from Amersham.

### Semi-quantitation of western blot results

After exposure, the X-ray films were analyzed with the Automated Digitizing System from the Silk Scientific Corporation (Orem, UT) as previously described [21]. The relative expression levels (fold) were calculated by dividing the averaged total pixel (from three experiments) for each band under investigation by the averaged total pixel for the corresponding β-actin band.

**Figure 2:**
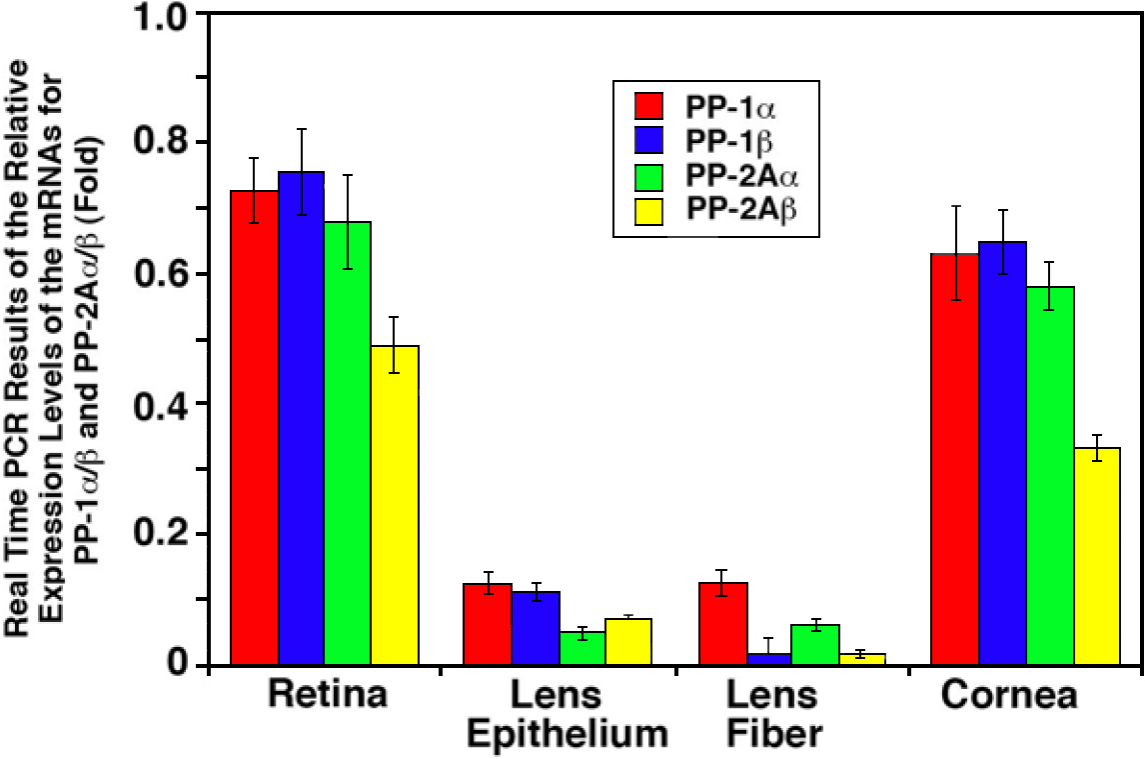
Real-time PCR to detect the mRNA levels for the catalytic subunits of PP-1α, PP-1β, PP-2Aα, and PP-2Aβ in the retina, lens epithelium, lens fiber, and cornea of the mouse eye. Real-time PCR procedures are described in the Methods section. The primers used in this study are listed in Table 2. Note that the mRNAs for the α and β catalytic subunits of both PP-1 and PP-2A were expressed at the highest levels in the retina and slightly reduced in the cornea but reduced 6–18 fold in the ocular lens of the mouse eye.

### Immunohistochemistry analysis

While numerous trial experiments (different fixation conditions and sectioning conditions) with adult mouse eyes failed to produce the intact sections of the mouse eyeballs, we turned to use a 15.5-day-old embryonic mouse eye for the immunohistochemistry analysis. The 15.5-day-old embryonic mouse eye was fixed in Bouin’s solution for less than 24 h. The fixed tissues were processed for dehydration and clearing and then embedded in paraffin wax according to Kaufman [22]. Five-micrometer thick sections were cut in a Leica RM2015 Microtome and then transferred to slides, which were processed for hematoxylin and eosin (HE) staining according to standard procedures [22].

For immunohistochemistry, the eye tissues were fixed with 4% paraformaldehyde in 0.1 M Sorensen’s phosphate buffer, pH 7.4 and embedded in paraffin. Five-micrometer thick sections were adhered to ChemMate capillary slides (Dako, Glostrup, Denmark). For antigen unmasking, sections were heated in 10 mM sodium citrate buffer (pH=6.0) for 1 min, and then the temperature of the sections was maintained at 95 °C for 9 min. Following the heating, the sections were cooled down to room temperature and then washed with distilled H_2_O three times (5 min each). The sections were quenched in 1% hydrogen peroxide for 10 min and then washed three times with distilled H_2_O and one time in PBS. For non-specific blocking, each section was incubated in 400 µl of normal goat serum (Jackson Immuno Research Laboratories, West Grove, PA) plus 0.1% Triton X-100 for 1 h at room temperature and then incubated overnight in 400 µl of diluted antibodies (1:100 to 1:400; see western blot analysis subsection for list of various antibodies) in a humidified chamber. The sections were then washed with PBS three times (5 min each) followed by incubation in 400 µl of secondary antibodies linked with FITC (1:1000 dilution in blocking solution) for 2 h in the absence of visible light. After incubation, sections were washed with PBS at room temperature six times (5 min each) and then observed under a Nikon fluorescence microscope (Model Y-FL 075042; Nikon Inc., Melville, NY). For negative controls, the sections were treated in the same way except that the primary antibody was replaced with normal serum.

**Figure 3:**
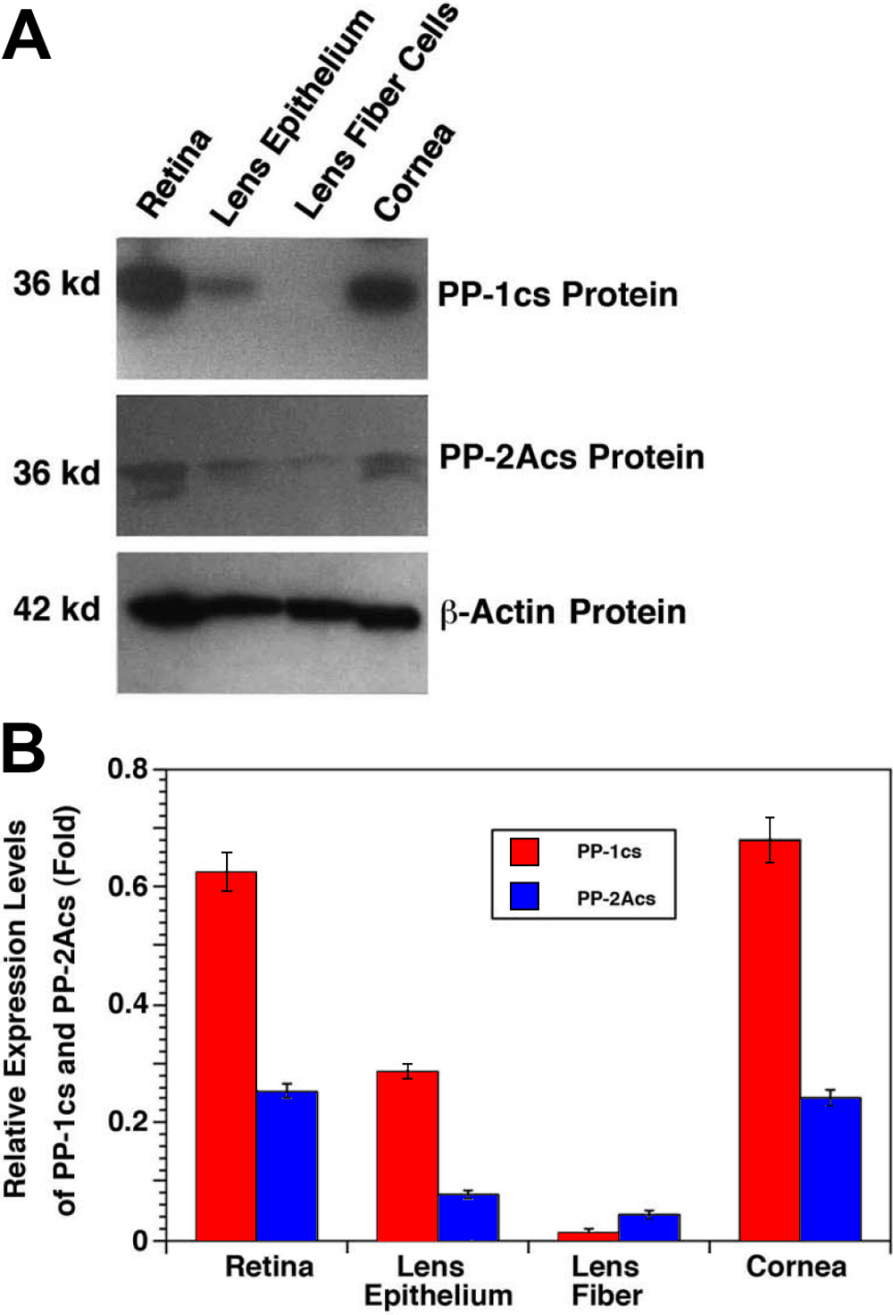
Western blot analysis of the protein for the catalytic subunits of PP-1 (PP-1cs) and PP-2A (PP-2Acs) in the retina, lens epithelium, lens fiber, and cornea of the mouse eye. **A**: Fifty micrograms of total proteins extracted from different ocular tissues of the mouse eye were separated by 10% SDS–PAGE, transferred to nitrocellulose membranes, and probed with an antibody recognizing both α and β isoforms of PP-1cs (top panel) or an antibody recognizing both α and β isoforms of PP-2Acs (middle panel) or an antibody recognizing β-actin (bottom panel) at 1:1000 dilution for 60 min. After three washes with TBS-T (10 mM Tris, pH 8.0; 150 mM NaCl, 0.05% Tween 20), the blots were incubated for 45 min with anti-mouse IgG (1:1000 dilution), which was linked to peroxidase. At the end of the incubation, the blots were washed twice with TBS-T followed by another two washes with TBS (10 mM Tris, pH 8.0; 150 mM NaCl) and finally visualized with the Amersham ECL kit. Note that the PP-2Acs in most cases appear in two bands due to different phosphorylation status. Molecular weight was determined according to the Bio-Rad (Hercules, CA).protein standard. **B**: Quantitative results of the expression of PP-1 (PP-1cs) and PP-2A (PP-2Acs) in the retina, lens epithelium, lens fiber, and cornea of the mouse eye are shown in the chart. After exposure, the bands on each X-ray film were processed with the Automated Digitizing System from the Silver Scientific Corporation. The relative level of expression (fold) was calculated by dividing the averaged total pixel (from three experiments) for each band with the averaged total pixel from the corresponding β-actin band. Note that in the retina, lens epithelium, and cornea, PP-1cs is more predominant than PP-2Acs. Since such a difference was not so obvious at the mRNA levels, differential translation or stability of PP-2Acs may exist.

### Cell culture and expression constructs

The αTN4–1 cells were grown in Dulbecco’s Modified Eagle’s Minimal Essential Medium (DMEM) containing 10% fetal bovine serum and 50 units/ml penicillin and streptomycin as described before [16,17]. All cells were kept at 37 °C and 5% CO_2_ gas phase. The expression constructs, pCI-neo, pCI-neo-PP1α, and pCI-neo-PP2Aα, were previously described [17,23]. The αTN4–1 cells that stably transfected with pCI-neo vector, pCI-neo-PP-1α, and pCI-neo-PP2Aα were first screened for four weeks with Enger's Minimal Essential Medium (EMEM) containing 10% fetal bovine serum and 50 units/ml penicillin and streptomycin plus G418 medium (400 µg/ml). After screening, individual stable clones were grown in the same medium.

### MTT assays for cell viability

The stable clones were grown to 100% confluence in EMEM containing 10% fetal bovine serum and 50 units/ml penicillin and streptomycin plus G418 medium (400 µg/ml). The different clones of cells were passaged into 96 well plates at a density of 2.5x10^4^ cells/per well and allowed to grow for 12 h in the same medium. Then, the different clones of cells were incubated in serum-free EMEM containing 50 units/ml penicillin and streptomycin plus G418 medium (400 µg/ml) for 2 h. By the end of the 2 h incubation, the medium was replaced with the same serum-free medium containing 100 µM H_2_O_2_, 50 mU glucose oxidase, and 19 µM glucose. Under this condition, the medium maintains 85–95 µM hydrogen peroxide within the period of treatment (0–6 h). By the end of the treatment (0 h, 3 h, and 6 h), the medium containing H_2_O_2_ was removed, and 100 µl MTT [3-(4,5-dimethythiazol-2-yl)-2,5diphenyltetrazolium bromide)] solution (100 µg/ml) was added into each cell culture well and incubatied for 6 h. At the end of MTT incubation, the supernatant was removed, and 100 µl DMSO were added into each well to dissolve the crystal with a 5 min gentle shake. After the crystal was dissolved, the plate was read at 490 nM in a Beckman Spectrophotometer. The relative cell death was compared among the stable clones transfected with pCI-neo vector, pCI-neo-PP-1α, and pCI-neo-PP2Aα.

**Table 2:** Oligo Primers used for real-time PCR studies.

**Primer name**	**Oligo primer sequence**
PP-1α (+)	5′-ATCCTCAAGCCCGCTGATAAGA-3′
PP-1α (−)	5′-GCCACTGAACTGCCCATACTTC-3′
PP-1β (+)	5′-GGTCCGACCCAGATAAGGATG-3′
PP-1β (−)	5′-TGATGAGCTCGACAAATCAAGTCTA-3′
PP-2Aα (+)	5′-CATGAGGGTCCAATGTGTGACT-3′
PP-2Aα (−)	5′-ACCACGGTCATCTGGATCTGA-3′
PP-2Aβ (+)	5′-TGTAGCGTTAAAGGTGCGCTATC-3′
PP-2Aβ (−)	5′-GGCACTCATCATAAAAGCCATACAC-3′
β-actin (+)	5′-GTACGTAGCCATCCAGGCTGTGT-3′
β-actin (−)	5′-GTGGTACGACCAGAGGCATACA-3′
PP2A-Aα (+)	5′-TGACGAGCAGGACTCGGTG-3′
PP2A-Aα (−)	5′-TGTAGCGAACACGCCAAGAC-3′
PP2A-Aβ (+)	5′-AGAATGACCACGCTCTTCTGC-3′
PP2A-Aβ (−)	5′-TTGCTACCTGGTCTCCTGCC-3′
PP2A-Bα (+)	5′-GAAGAACCTGAAGATCCCAGCA-3′
PP2A-Bα (−)	5′-CACGGGCCTGTTCTCCATA-3′
PP2A-Bβ (+)	5′-ACAATGTTTACAGCACATTCCAGAG-3′
PP2A-Bβ (−)	5′-GCTTCACAGTTTTATCGTTGGTAGAC-3′
PP2A-Bγ (+)	5′-AAGCAGCCTGGCCTCATCT-3′
PP2A-Bγ (−)	5′-TCCTCGCTGTCAAACAGCTCTA-3′

### Detection of phospho-p53 and Bak expression

The detection of p53 hyperphosphorylation at Serine-15 and Bak upregulation were conducted as previously described [23].

## Results

### mRNA expression of PP-1α/β and PP-2Aα/β catalytic subunits

We have previously analyzed the expression patterns of the mRNA for the catalytic subunits of PP-1α and PP-2Aα in both epithelial and fiber cells of bovine and rat lenses [13]. To further explore the expression patterns of these phosphatases in the eye, we compared the expression patterns of both α and β catalytic subunits for PP-1 and PP-2A in the retina and cornea along with the lens epithelium and lens fiber cells in the mouse eye. First, we conducted reverse transcription polymerase chain reaction (RT–PCR) analysis. For PP-1α mRNA expression, a strong band of 397 bp from the PP-1α specific primers was detected in the retina, a reduced level of the same band was observed in the cornea, and a much reduced level of the same band was found in lens epithelium and fiber cells (Panel 1 of Figure 1). A similar expression pattern of PP-1β was observed in the retina, lens epithelium, and cornea of the mouse eye, but the amplified 463 bp PP-1β band was absent in the lens fiber cells (Panel 2 of Figure 1). For PP-2Aα, the mRNA expression pattern was also similar to that of PP-1α except that the 468 bp PP-2Aα band was just barely detectable in the lens epithelial cells (Panel 3 of Figure 1). For PP-2Aβ, the mRNA expression pattern is similar to that of PP-1β. The highest level of PP-2Aβ was found in the retina, and a much-reduced level of PP-2Aβ was detected in the cornea (Panel 4 of Figure 1). In the ocular lens, PP-2Aβ was detectable in the lens epithelium but absent in the lens fiber cells (Panel 4 of Figure 1).

To further confirm the mRNA expression patterns of the α and β catalytic subunits for PP-1 and PP-2A, we conducted real time PCR analysis. As shown in Figure 2 of the mouse retina, the mRNA level for PP-1β was slightly higher than that of PP-1α, and the mRNA for the PP-2Aα was about 40% higher than that of PP-2Aβ. Consistent with the results of RT–PCR (Figure 1), the real-time PCR confirmed that both α and β subunit mRNAs of PP-1 and PP-2A displayed the highest levels of expression in the mouse retina and slightly reduced in the mouse cornea. However, in the ocular lens of the mouse eye, the expression levels of PP-1α and PP-1β were reduced six to sevenfold compared with those in the retina and cornea (Figure 2). Real-time PCR also confirmed that PP-1β and PP-2Aβ were absent in the mouse lens fiber cells and PP-2Aα was just barely detectable in the lens epithelial cells (Figure 2).

**Figure 4:**
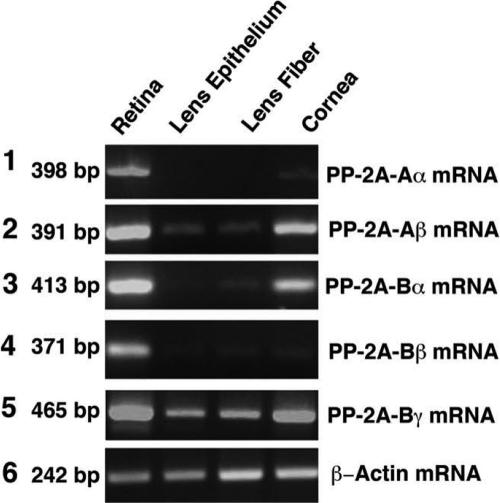
RT–PCR to detect the mRNA levels for the regulatory subunits of PP2A-Aα, PP2A-Aβ, PP2A-Bα, PP2A-Bβ, and PP2A-Bγ in the retina, lens epithelium, lens fiber, and cornea of the mouse eye. RT–PCR was conducted as described in Figure 1. The primers used in the present studies are described in Table 1. The primers for each PP-2A regulatory subunit and for β-actin were added to the reactions at the same time. The 398 bp DNA band for the PP2A-Aα mRNA, the 391 bp DNA band for PP2A-Aβ, the 413 bp DNA band for PP2A-Bα, the 317 bp DNA band for PP2A-Bβ, and the 465 bp DNA band for PP2A-Bγ mRNA were all expressed at the highest levels in the retina, at reduced levels (PP2A-Aβ, PP2A-Bα, and PP2A-Bγ) or barely detectable levels (PP2A-Aα and PP2ABβ) in the cornea, and at much reduced levels (PP2A-Aβ and PP2A-Bγ), barely detectable levels (PP2A-Bα and PP2A-Bβ), or absent (PP2A-Aα) in the lens epithelium or fiber cells. As an internal control, a β-actin DNA band of 242-bp is also amplified.

### Expression of PP-1cs and PP-2Acs proteins in the retina, lens epithelium, lens fiber, and cornea of the mouse eye

Both RT–PCR and real-time PCR reveal that mRNA for the two catalytic subunits of either PP-1 or PP-2A is differentially expressed in four different ocular tissues of mouse eye. To explore whether similar expression patterns exist for the catalytic subunit proteins of PP-1 and PP-2A, we conducted western blot analysis. As shown in Figure 3, the two catalytic subunits for both PP-1 and PP-2A in the cornea are slightly higher than those in the retina, a pattern opposite to that for the corresponding mRNA expression (Figure 1 and Figure 2). On the other hand, the much reduced expression patterns of the catalytic subunits for both PP-1 and PP-2A in the lens epithelium and lens fibers parallel their expression patterns of the mRNA (Figure 3, also see Figure 1 and Figure 2). Western blot analysis also reveals another differential expression feature between PP-1cs and PP-2Acs. Although the mRNA for the two catalytic subunits of PP-2A is only slightly lower than those of PP-1 in all four ocular tissues (Figure 1 and Figure 2), the catalytic subunit proteins for PP-2A are much lower than those of PP-1 (Figure 3), clearly indicating the presence of differential translation or stability of PP-2A catalytic subunits.

**Figure 5:**
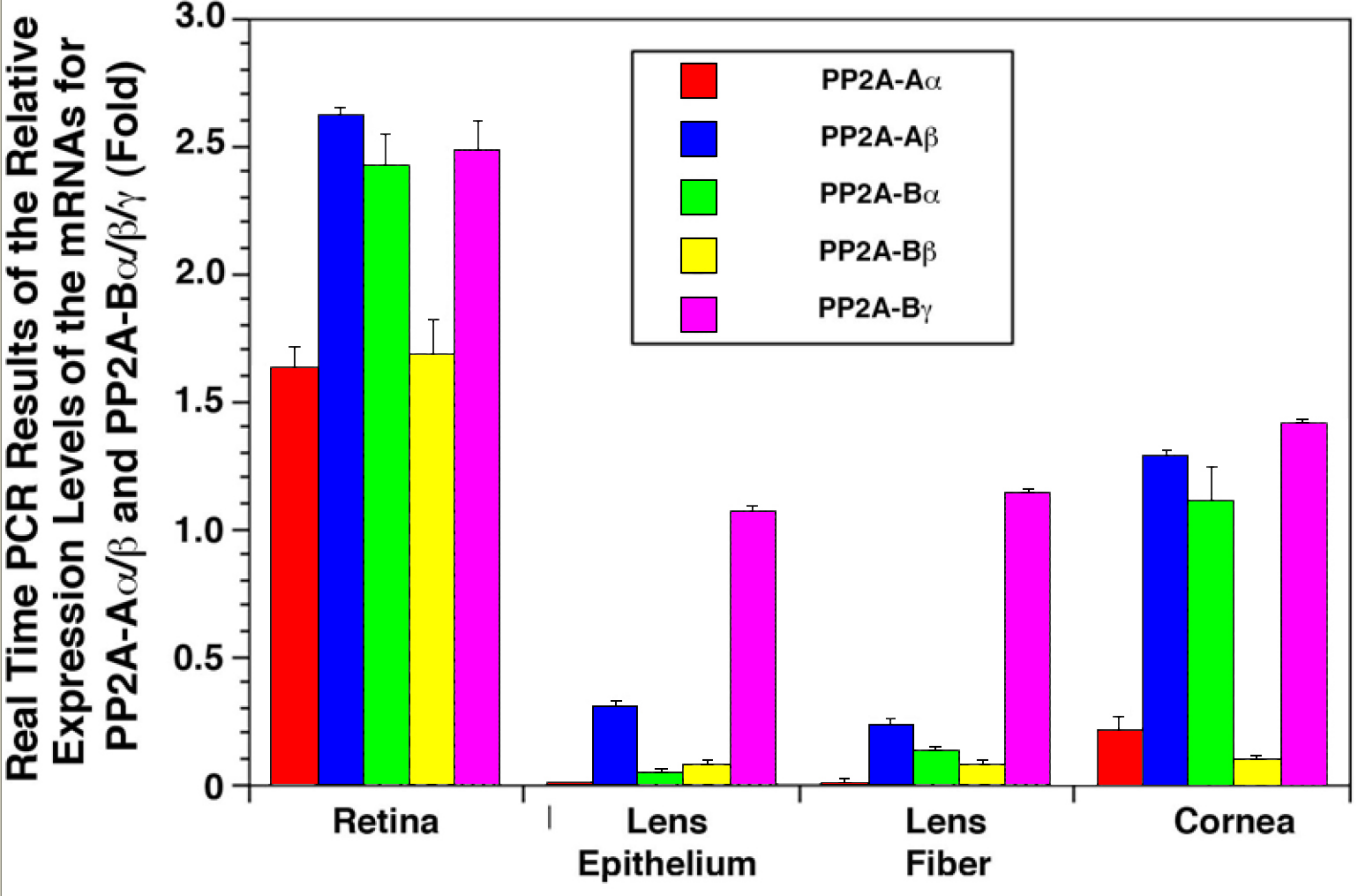
Real-time PCR to detect the mRNA levels for the regulatory subunits of PP2A-Aα, PP2A-Aβ, PP2A-Bα, PP2A-Bβ, and PP2A-Bγ in the retina, lens epithelium, lens fiber, and cornea of the mouse eye. Real-time PCR procedures are described in the Methods section. The primers used in this study are listed in Table 2. Note that the real-time results confirm the RT–PCR patterns of mRNA levels for the regulatory subunits of PP2A-Aα, PP2A-Aβ, PP2A-Bα, PP2A-Bβ, and PP2A-Bγ in the retina, lens epithelium, lens fiber and cornea of the mouse eye.

### mRNA expression of PP2A-Aα/β and PP2A-Bα/β/γ in the mouse eye

Since the functions of PP-2A are largely determined by its regulatory subunits, PP2A-A and PP2A-B [7], we next analyzed their mRNA expression patterns in different tissues of the mouse eye. The mRNA for both α and β isoforms of PP2A-A subunits in the mouse retina displayed the highest levels of expression among the retina, lens epithelium, lens fiber, and cornea of the mouse eye (Panels 1 and 2 of Figure 4). While PP2A-Aα mRNA was detectable in the cornea, this scaffold subunit of PP-2A was completely absent in the ocular lens. In contrast, the PP2A-Aβ scaffold subunit mRNA was strongly expressed in the cornea and easily detected in the lens epithelium and lens fibers (Panels 1 and 2 of Figure 4). The mRNAs for three isoforms, α, β, and γ, of PP2A-B subunits in the retina also displayed the highest levels of expression among the four different ocular tissues of mouse eye (Panels 3–5 of Figure 4). In the cornea, while PP2A-Bα and PP2A-Bγ mRNAs were strongly expressed, PP2A-Bβ mRNA was just barely detectable (Panels 3–5 of Figure 4). In the ocular lens, only PP-2A-Bγ mRNA was prominently detected (Panel 5 of Figure 4). Both PP2A-Bα and PP2A-Bβ were barely detectable in the lens fiber cells and the lens epithelium (Panels 3 and 4 of Figure 4).

Real-time PCR analysis of the mRNAs for PP2A-Aα, PP2A-Aβ, PP2A-Bα, PP2A-Bβ, and PP2A-Bγ confirmed the general expression patterns of the five mRNAs revealed by RT–PCR (Figure 5). Moreover, it was found that in the mouse retina, the mRNA level for PP-2Aβ was approximately 0.62 fold more than that for PP-2Aα. Among the three PP2A-B subunits in retina, the mRNAs for both PP2A-Bα and PP2A-Bγ were about 0.5 fold more than that for PP2A-Bβ. In the cornea, both PP2A-Bα and PP2A-Bγ were present but the levels of the two mRNAs were reduced to half compared with those found in retina (Figure 5). In the ocular lens, the PP2A-Aα mRNA was present with a level of about 7.6 fold to eightfold less than found in the retina (Figure 5). Similarly, PP2A-Bγ was present with a level of about 1.3 fold less than found in the retina (Figure 5).

### Protein expression of PP2A-Aα/β and PP2A-Bα/β/γ in mouse eyes

**Figure 6:**
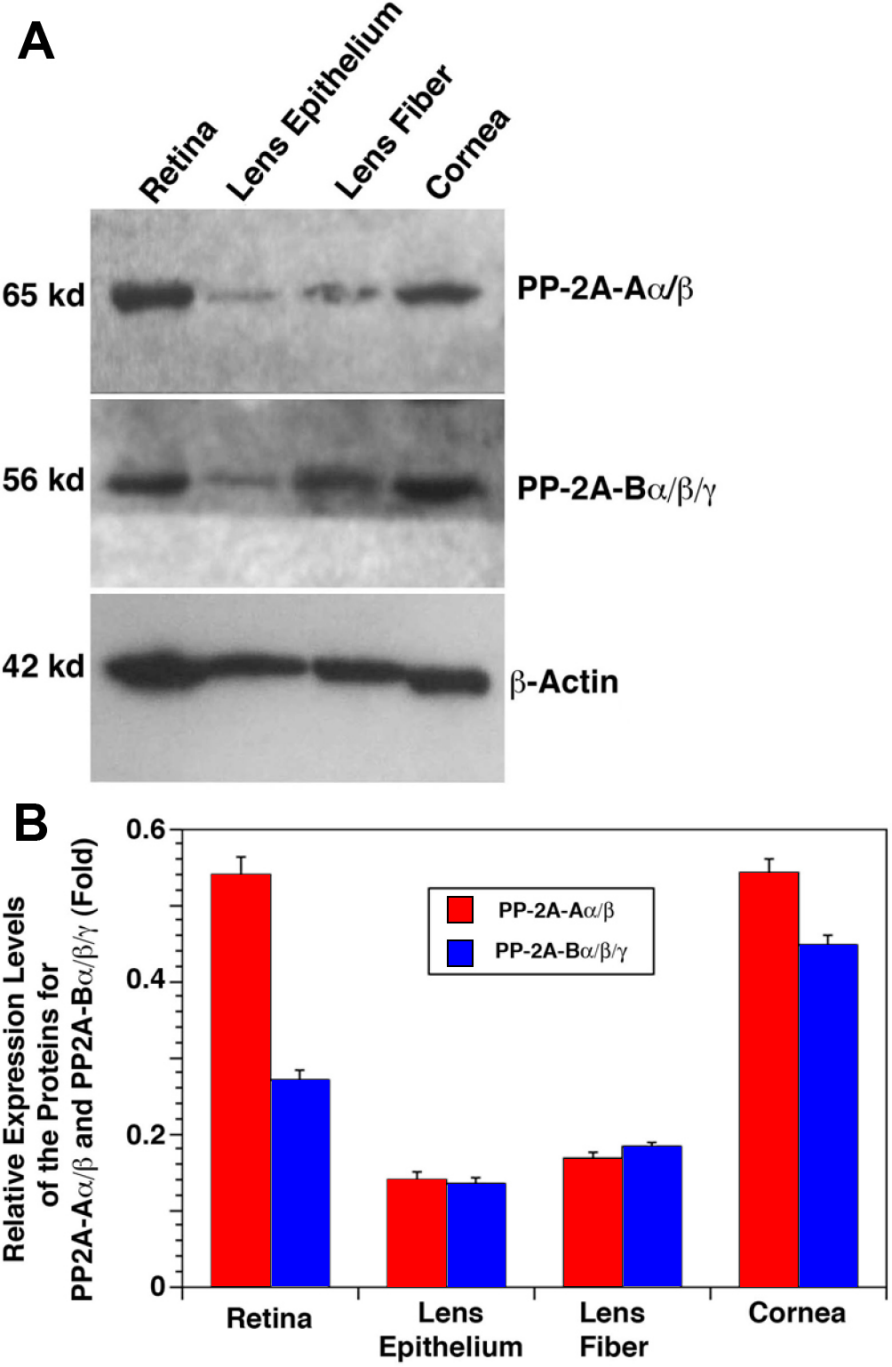
Western blot analysis of the proteins for the regulatory subunits of PP2A-A and PP2A-B in the retina, lens epithelium, lens fiber, and cornea of the mouse eye. **A**: Fifty micrograms of total proteins extracted from different ocular tissues of the mouse eye were separated by 10% SDS–PAGE, transferred to nitrocellulose membranes, and probed with an antibody recognizing both α and β isoforms of PP2A-A subunit (top panel) or an antibody recognizing all α, β and γ isoforms of PP2A-B subunit (middle panel) or an antibody recognizing β-actin (bottom panel) at a dilution of 1:500 (for PP-2A regulatory subunits) or 1:1000 (for β-actin) for 60 min. After three washes with TBS-T (10 mM Tris, pH 8.0; 150 mM NaCl; 0.05% Tween 20), the blots were incubated for 45 min with anti-mouse IgG (1:1000 dilution), which was linked to peroxidase. At the end of the incubation, the blots were washed twice with TBS-T followed by another two washes with TBS (10 mM Tris, pH 8.0; 150 mM NaCl) and finally visualized with the Amersham ECL kit. The molecular weight was determined according to the Bio-Rad protein standard. **B**: Quantitative results of the expression of the regulatory subunits of PP2A-A and PP2A-B in the retina, lens epithelium, lens fiber, and cornea of the mouse eye are shown in the chart. After exposure, the bands on each X-ray film were processed with the Automated Digitizing System from the Silver Scientific Corporation. The relative level of expression (fold) was calculated by dividing the averaged total pixel (from three experiments) for each band with the averaged total pixel from the corresponding β-actin band. Note that the two regulatory subunits, A and B, of PP-2A were predominantly expressed in the retina and cornea and at substantially reduced levels in the lens. In addition, expressions of the two regulatory subunits for PP-2A were slightly higher in lens fibers than in lens epithelial cells.

To further explore the expression patterns of the regulatory subunits for PP-2A, we conducted western blot analysis. Our initial preliminary studies revealed that only two antibodies yielded positive results in our western blot analysis. One antibody recognized α and β isoforms of PP2A-A and another antibody recognized α, β, and γ isoforms of PP2A-B. Both antibodies were obtained from Santa Cruz Biotechnology and were used for further experimentation. As shown in Figure 6, the proteins from both PP2A-A and PP2A-B subunits were highly expressed in the mouse retina and cornea. The level of PP2A-Aα/β in the retina and cornea displayed similar levels. However, the PP2A-Bα/β/γ level in the retina was about 0.6 fold less than that in the cornea (Figure 6B). In the ocular lens, expression of the two regulatory subunits for PP-2A was substantially reduced in the lens fiber cells, and such reduction was even more in the lens epithelium (Figure 6A). Semi-quantitative analysis of these regulatory subunits in the four ocular tissues demonstrated that the level of PP2AAα/β was reduced 2.9 fold in the lens epithelium and 2.1 fold in the lens fiber cells compared with its expression level in the retina (Figure 6B). PP2A-Bα/β/γ was also reduced onefold in the lens epithelium and reduced 0.75 fold in the lens fiber cells in comparison to its level in the retina (Figure 6B)

### Immunohistochemical analysis of PP2A-Aα/β and PP2A-Bα/β/γ in the mouse eye

**Figure 7:**
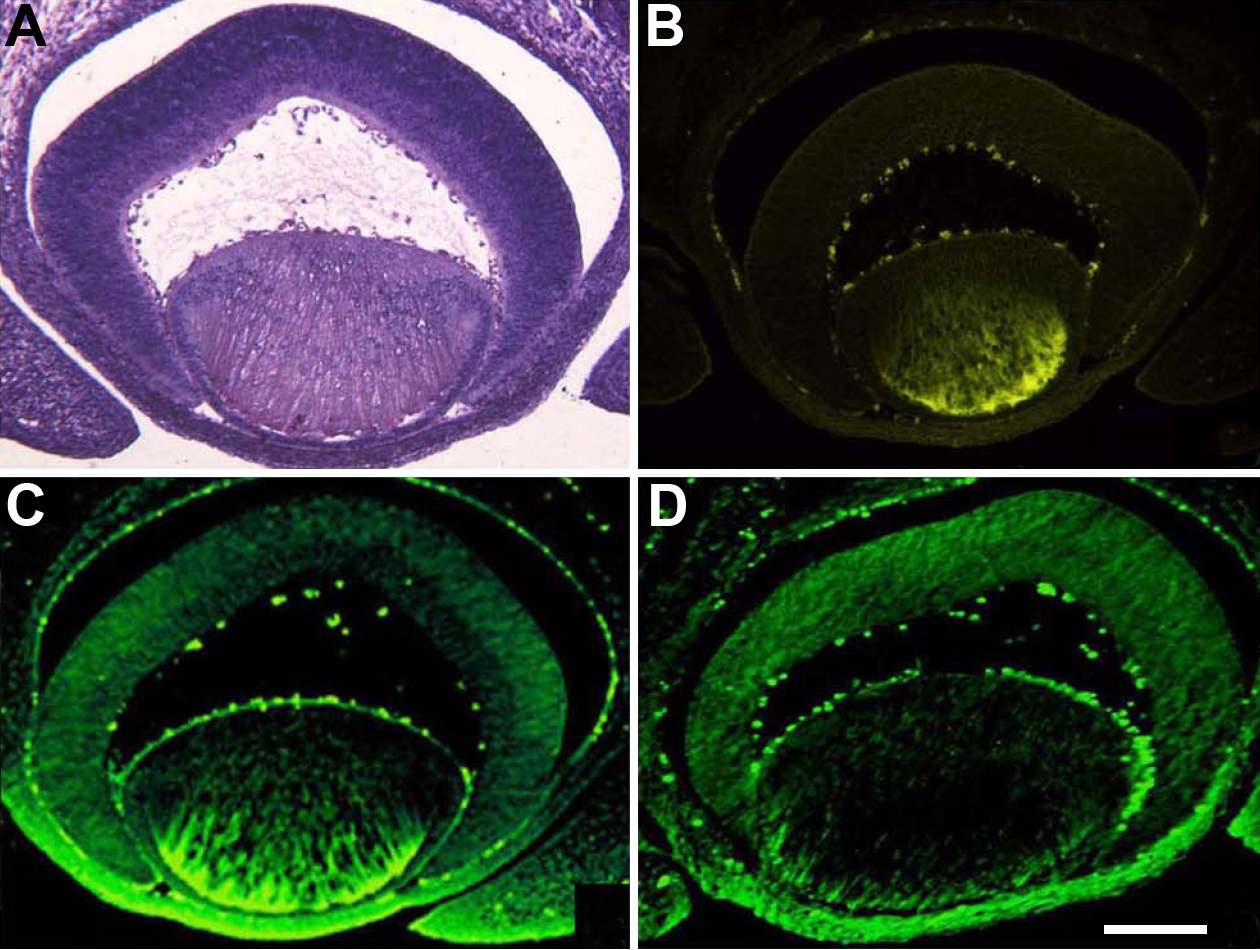
Immunohistochemical analysis of the proteins for the regulatory subunits of PP2A-A and PP2A-B in the retina, lens epithelium, lens fiber and cornea of 15.5-day-old mouse eye. The experimental procedures are described in the Methods section. The two antibodies used for western blot analysis in Figure 6 were also used here in immunohistochemical analysis. Note that both A and B subunits of PP-2A were strongly expressed in the cornea. In the retina, the B subunit is homogenously expressed in both the neuro-retina layer and pigment retina layer. In contrast, the expression of the regulatory A subunits is stronger in the pigment layer than in the neuro-retina layer. Expression of both A and B subunits of PP-2A seems stronger in the fiber cells than in the lens epithelium. In addition, while the A subunit appears in both the central and cortical fibers, B subunit is mostly distributed in the cortical fibers. **A**: hematoxylin and eosin (HE) staining of the section cut from15.5-day-old mouse eye. **B**: The section cut from the same embryonic mouse eye of **A** was used for immunohistochemistry analysis with normal serum. **C**: The section from the same embryonic mouse eye of **A** was used for immunohistochemistry staining with anti-PP2A-Aα/β antibody. Note that PP2A-Aα/β expression was different in the two layers of retina. **D**: The section from the same embryonic mouse eye of **A** was used for immunohistochemistry staining with anti-PP2A-B α/β/γ antibody. Note that PP2A-Bα/β/γ expression was homogenous in both layers of retina.. Bar: 0.2 mm.

To determine the localization of the regulatory subunit A and B of PP-2A in the mouse eye, we conducted immunohistochemical analysis on these regulatory subunits. As shown in Figure 7C, the most intensive immunofluorescence signal of PP2A-Aα/β was observed in the entire cornea. The second strongest immunofluorescence signal was detected in the pigment layer of the mouse retina. The immunofluorescence signal in the neuro-retinal layer of the mouse retina is about the same as that in the lens fiber cells. The lowest fluorescence signal was found in the lens epithelial cells (Figure 7C). For PP-2Αα/β/γ, the most intensive fluorescence signal was also found in the cornea (Figure 7D). Different from the staining pattern of PP2A-Aα/β, PP2A-Bα/β/γ was homogenously distributed in both layers of the mouse retina (Figure 7D). In the ocular lens, a narrow band of anterior cortical fiber cells were stained very strongly for PP2A-Bα/β/γ. In the nuclear fiber, the fluorescence signal for PP2A-Bα/β/γ was weakly stained or absent (Figure 7D). In summary, the immunocytochemical analysis confirmed the results from western blot analysis that both A and B regulatory subunits of PP-2A were highly expressed in the cornea and retina but substantially reduced in the ocular lens.

### PP-1 and PP-2A protect mouse lens epithelial cells from oxidative stress-induced cell death

**Figure 8:**
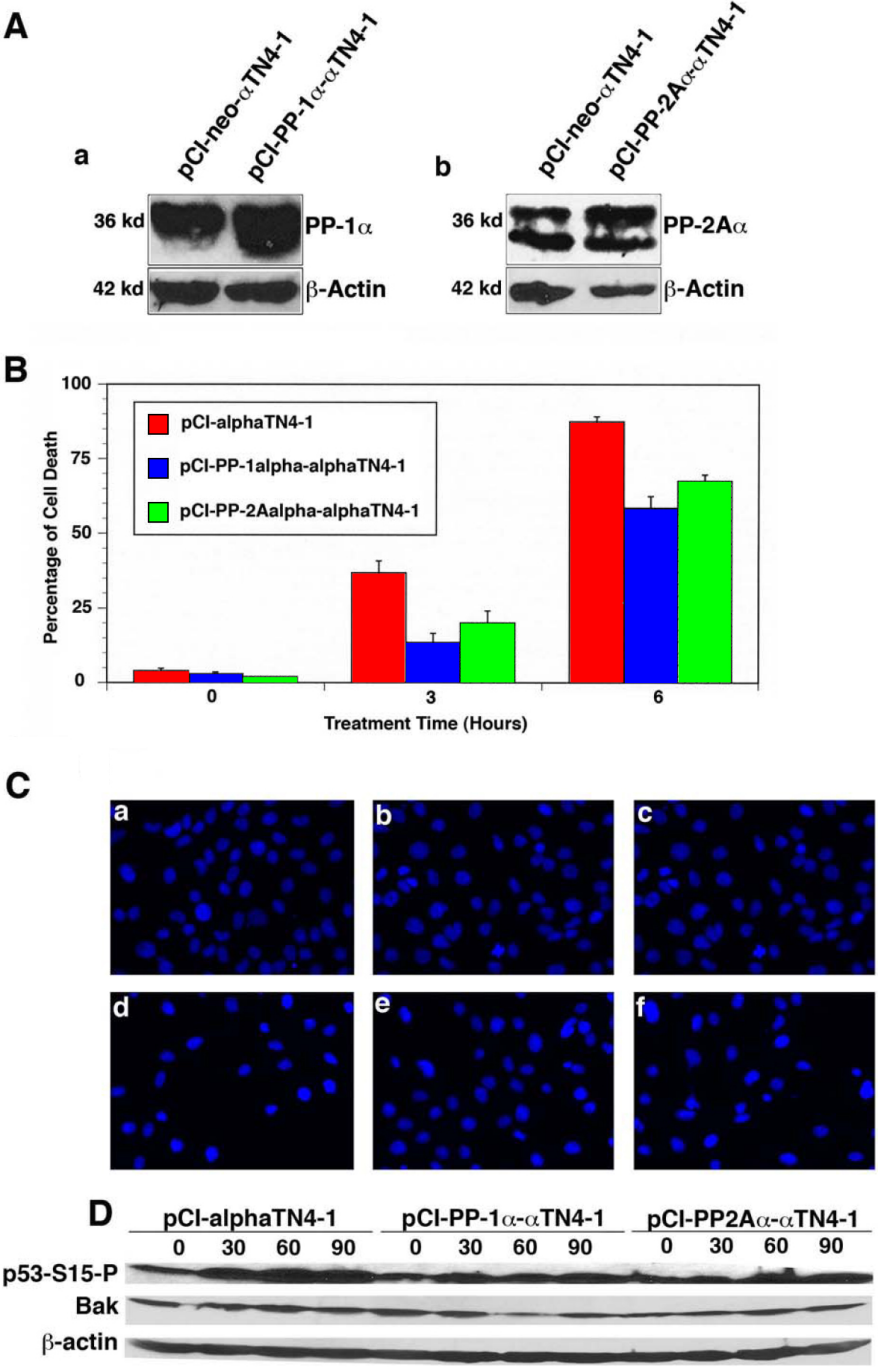
PP-1 and PP-2A protect lens epithelial cells from oxidative stress-induced apoptosis. **A**: western blot analysis confirms the overexpression of PP-1α (a) and PP2Aα (b) in αTN4–1 cells. The stable clones expressing the pCI-neo vector, pCI-PP-1α, or pCI-PP-2Aα were obtained through G418 screen (400 µg/ml). The expression levels of PP-1α and PP-2Aα were determined by western blot analysis using 100 µg of total protein extracted from pCI-neo or PP-1α-tranfected αTN4–1 cells (a) and from pCI-neo or PP-2Aα-transfected αTN4–1 cells (b). **B**: Results of the MTT assay is shown in the chart. The MTT assay is described in the Methods section. Note that PP-1α displayed a stronger ability against oxidative stress-induced cell death than PP-2Aα did. **C**: Hoechst staining analysis of the pCI-neotransfected cells (a, d), PP-1α-tranfected αTN4–1 cells (b, e), or PP-2Aα-transfected αTN4–1 cells (c, f) without treatment (a, b, c) or treated by 85–95 µM H2O2 (d, e, f) for 3.5 h is pictured. Hoechst staining was conducted as previously described [14,17]. Note that after treatment, the apoptotic cells became either dissociated from the culture plate (thus leaving empty space in the culture dish) or condensed. **D**: Western blot analysis of the p53 hyperphosphorylation at Ser-15 and Bak expression. The western blot analysis was conducted as described before [23]. Note that in both PP-1α and PP-2Aα-transfected cells, hydrogen peroxide-induced hyperphosphorylation of p53 at Ser-53 and Bak upregulation were obviously attenuated.

To assay the function of PP-1 and PP-2A in the mouse eye, we have transfected the mouse lens epithelial cell line αTN4–1 with the constructs expressing PP-1α or PP-2Aα. The stable cones were screened in the presence of 400 µg/ml G418. The overexpression of PP-1α and PP-2Aα was confirmed with western blot analysis (Figure 8A). When the stable clones of αTN4–1 cells transfected with pCI-neo vector alone, pCI-PP-1α, or pCI-PP-2Aα expression constructs [17,23] were treated with 85–95 µM H_2_O_2_ for 0–6 h, MTT assays revealed that PP-1α and PP-2Aα displayed differential protection against oxidative stress-induced cell death (Figure 8B). The apoptotic nature induced by H_2_O_2_ was confirmed by Hoechst staining analysis (Figure 8C). The apoptotic pathway activated by H_2_O_2_ includes the activation of p53 and upregulation of the proapoptotic gene, *Bak* (Figure 8D), a direct target of p53 as we recently demonstrated in mouse JB6 cells [23]. The PP-1α- and PP-2Aα-transfected cells clearly displayed lower levels of p53 hyperphosphorylation at Serine-15 and Bak upregulation induced by H_2_O_2_.

## Discussion

In the present communication, we have investigated the expression patterns of the genes encoding the catalytic subunits for PP-1 and PP-2A and their functions against oxidative stress-induced cell death as well as the expression patterns of the regulatory subunits, A and B, of PP-2A. In all four ocular tissues examined, the catalytic subunits for PP-1 (PP-1cs) are predominantly expressed at both mRNA and protein levels over the catalytic subunits for PP-2A (PP-2Acs). Among the four tissues, the mRNAs for the α and β catalytic subunits of PP-1 and PP-2A displayed the highest levels of expression in the retina and slightly reduced levels in the cornea but much reduced levels in the ocular lens of the mouse eye. The proteins for the catalytic subunits of PP-1 and PP-2A were strongly expressed in both the retina and cornea but substantially reduced in the lens epithelium and barely detectable (PP-2Acs) or absent (PP1cs) in lens fiber cells of the mouse eye. The mRNAs for five isoforms (PP2A-Aα/β and PP2A-Bα/β/γ) of PP2A-A and PP2A-B subunits are highly expressed in the mouse retina, but three out of the 5 mRNAs are reduced and the remaining two barely detectable in mouse cornea. In the ocular lens of the mouse eye, only the mRNAs for PP2A-Aβ and PP2A-Bγ are clearly detectable in both the epithelial and fiber cells. The proteins for both A and B subunits are highly expressed in the retina and cornea but are much less abundant in the ocular lens. Moreover, the lens fiber cells have slightly higher levels of PP2A-Aα/β and PP2A-Aα/β/γ proteins than the lens epithelial cells. The strongest fluorescence signals for PP2A-Aα/β and PP2A-Bα/β/γ proteins were detected in the mouse cornea, and somewhat reduced signals were detected in the retina and ocular lens. Also, P2A-Aα/β but not PP2A-Bα/β/γ is differentially distributed in the mouse retina. Finally, both PP-1α and PP-2Aα are able to protect mouse lens epithelial cells from oxidative stress-induced apoptosis. PP-1α displayed stronger anti-apoptotic ability than PP-2Aα did. These results demonstrate that both PP-1 and PP-2A may play important roles in four different tissues of the mouse eye and that the function of PP-2A seems to be highly regulated in these ocular tissues. Indeed, various functions of the protein serine/threonine phosphatases in the eye has recently begun to be unfolded [13-17,24-27].

First, the protein serine/threonine phosphatases are involved in regulation of eye development. For example, during drosophila eye development, the catalytic subunit of PP-2A is found to be implicated in the regulation of the R7 photoreceptor specification through both positive and negative regulation of the MEK/ERK signaling pathway [25]. Wassarman et al. [25] have demonstrated that a reduction in the dose of the gene encoding the catalytic subunit for PP-2A stimulates the signal from Ras1 but impairs the signal from Raf.

Regulation of eye development by protein phosphatases is also observed in the vertebrate. In *Xenopus*, PP-2A is involved in the regulation of eye induction and subsequently eye field separation. Rorick et al. [27] have shown that one of the B family subunits for PP-2A is required for the IGF/PI3K/Akt pathway and that interfering with the PI3K/Akt pathway inhibits eye induction. Furthermore, during eye field separation, this subunit is also implicated in regulating the hedgehog-signaling pathway [27].

Second, the protein serine/threonine phosphatases are implicated in the normal function of the eye. Edwards et al. [27] have reported that in the horseshoe crab, inhibition of PP-1 and PP-2A activity by okadaic acid interferes with physiologic functions of the photoreceptor cells. In the ocular lens, α-crystallins are important lens proteins, which act as molecular chaperones [28], anti-apoptotic regulators [29-31], and autokinases [32]. Previous studies from several laboratories have revealed that α-crystallins are phosphorylated by different serine/threonine kinases [33-35] and dephosphorylated by serine/threonine phosphatases including PP-2B [36] and other unknown phosphatases. A change of the phosphorylation status is closely linked to its functional ability. Hoover et al. [37] have demonstrated that phosphorylation of the serine residue at position 59 in αB-crystallin is critical for its cellular protection.

Finally, changes in the protein serine/threonine phosphatase expression or activity are implicated in the pathogenesis of the vertebrate and human eyes. In this regard, Palczewski et al. [38] have observed that rd mice have elevated PP-2A activity from postnatal day 5 to day 10. The loss of photoreceptors in rd mice begins on postnatal day 11. It is suggested that the elevated PP-2A activity may be implicated in photoreceptor death. In the murine model of glaucoma, Huang et al. [39] found that activation of PP-2B by increased intraocular pressure leads to apoptotic retinal ganglion cell death. Kantorow et al. [40] observed that the mRNA for the regulatory subunit of PP-2A is downregulated in the epithelial cells of the human cataractous lenses, suggesting possible roles of PP-2A activity in maintaining normal lens transparency. Consistent with this early observation, we demonstrate here that both PP2A-Aβ and PP2A-Bγ are expressed at higher levels in the fiber cells than in the epithelial cells (Figure 6). We have previously demonstrated that inhibition of the PP-1 activity by okadaic acid leads to upregulated expressions of the pro-apoptotic genes, which leads to eventual apoptosis of the treated lens epithelial cells [14,15]. Consistent with these early observations, we showed here that both PP-1 and PP-2A are able to protect mouse lens epithelial cells from oxidative stress-induced apoptosis (Figure 8B). The mechanisms mediating this protection include the attenuation of p53 hyperphosphorylation at Ser-15 as well as some other residues by PP-1 and PP-2A and also the attenuation of Bak upregulation (Figure 8D). Our recent studies have shown that both PP-1 and PP-2A are able to directly dephosphorylate p53 at these residues [16,23]. Stress-induced apoptosis of lens epithelial cells play an important role for non-congenital cataractogenesis [41-45].

In the present study, we demonstrated that the genes encoding both α and β catalytic subunits for PP-1 and PP-2A are highly expressed at the mRNA and protein levels in both the retina and cornea but are reduced in the ocular lens of the mouse eye. Such expression patterns likely reflect the difference of the functional requirements for these phosphatases by different eye tissues. In the retina, light-activated signaling pathways mediate rapid protein translocation within photoreceptors [38]. The constant transmission of the light-induced signals into the brain may need high levels of protein serine/threonine phosphatases to reverse the kinase action. The demonstration that the inhibition of PP-1 and PP-2A activity by okadaic acid blocks photoreceptor response in horseshoe crab [26] provides evidence to support this possibility. In the cornea, the epithelial cells are constantly renewed [46]. Since both PP-1 and PP-2A are involved in the control of the cell cycle [6], it is not surprising that highly renewal tissues such as the cornea have strong expression of both PP1 and PP-2A. In the ocular lens, on the other hand, cell division is relatively slow in the mature lens [47]. Although the lens epithelial cells at the equatorial region are constantly differentiating into new fiber cells, this process is significantly slowed in the mature lens [48]. Thus, it is not surprise that the expression levels of PP-1 and PP-2A are substantially reduced in the ocular lens than in the retina and cornea of the mouse eye (Figure 1 to Figure 3).

The holoenzyme of PP-2A consists of three different subunits, the regulatory A subunit, the catalytic C subunit, and the regulatory B subunit [7]. The A subunit serves as a scaffold to tether both C and B subunits. The B subunit is assembled into the A-C dimer of PP-2A after methylation of the C subunit [4-7], and the presence of strong expressions from all three B subunits (PP2A-Bα, PP2A-Bβ, and PP2A-Bγ) of PP-2A in the mouse retina suggests that PP-2A have multiple functions in this ocular tissue and such functions seem to be highly regulated. The relatively higher level of the PP2A-Aα/β protein distribution in the pigment layer than in the neuro-retina layer of the mouse retina (Figure 7C) suggests that the PP-2A function in the pigment layer may be even more important. In the mouse cornea, the two types of PP-2A regulatory subunits are also strongly expressed. However, different from the expression patterns of the PP-2A regulatory subunits in the mouse retina, both PP2A-Aα and PP2A-Bβ are much reduced.

Such results indicate that the functions of the scaffold PP2A-Aα and PP2A-Aβ subunits may be different, and such differential expression patterns of the PP2A-Aα and PP2A-Aβ subunits in the retina and cornea of the mouse eye may parallel the differential expression of the regulatory B subunits. In other words, certain members of the B regulatory subunits may have preferential affinity to PP2A-Aα than to PP2A-Aβ. This would also explain why in the ocular lens, both PP2A-Aα and PP2A-Bβ are also barely detectable or absent. Of course, different from both the retina and cornea, the ocular lens only expresses PP2A-Aβ and PP2A-Bγ, suggesting PP-2A function in the ocular lens may be less demanding. This would be consistent with our previous observation where the inhibition of PP-1 but not PP-2A caused apoptosis of lens epithelial cells [14,15].

In the present study, both RT–PCR and real-time PCR were used to determine the expression patterns of mRNA for genes encoding both catalytic subunits for PP-1 and PP-2A and also the regulatory subunits for PP-2A. While RT–PCR gave a direct observation of the amplified gene expression product, the real-time PCR presented a quantitative measurement. Our studies of the different mRNA levels with both methods demonstrated consistent results in majority of the samples of all four tissues (Figure 1, Figure 2, Figure 4, and Figure 5). Only a minor discrepancy was observed for the mRNA levels in the lens epithelium and lens fiber cells where expressions of the different mRNAs were lower than those in the retina and cornea. Such minor difference may be actually derived from the variations of the mRNA levels in different animals since the samples used for RT–PCR and real-time PCR were extracted from different groups of mice with the same age. Our results reveal that the expression levels of mRNAs for both α and β catalytic subunits of PP-2A are relatively lower than that of PP-1 in all for ocular tissues examined (Figure 1 and Figure 2). Such a difference is even more striking at the protein levels. In the retina, cornea, and lens epithelium of the mouse eye, the protein level of PP-1cs is twofold to threefold higher than that of PP2Acs (Figure 3). These results suggest that PP-1 is a more abundant protein serine/threonine phosphatase than PP-2A in the mouse eye. This result is consistent with our previous study where in both the rat and bovine lenses, we observed that PP-1cs is more abundant than PP-2A [13].

Although the two mRNAs for the α and β catalytic subunits of PP-1cs are only slightly higher than that of PP-2Acs, the proteins for α and β catalytic subunits of PP-1 are about twofold to fourfold higher than the corresponding catalytic subunits of PP-2A (Figure 3). These results suggest that the PP-2Aα/β mRNAs are either restricted during translation or PP-2Aα/β proteins are less stable. We are currently investigating the two possibilities. Regardless, such results clearly show that both PP-1cs and PP-2c are differentially expressed in the four ocular tissues of mouse eye.

In summary, our present studies provide important information regarding the expression patterns of the two most important protein serine/threonine phosphatases in different tissues of the mouse eye. Our results reveal that PP-1 is a more abundant phosphatase than PP-2A in the mouse eye and that the genes encoding both catalytic subunits for PP-1 and PP-2A and the regulatory subunits for PP-2A are all differentially expressed. These results provide a foundation for exploring the functions of PP-1 and PP-2A in the retina, cornea, and the ocular lens of the vertebrate eye. Furthermore, our results that a variety of regulatory subunits of PP-2A are all expressed in the retina and cornea of the mouse eye reveal that in these ocular tissues, PP-2A has highly regulated, important functions.
